# Dissecting Low Atmospheric Pressure Stress: Transcriptome Responses to the Components of Hypobaria in Arabidopsis

**DOI:** 10.3389/fpls.2017.00528

**Published:** 2017-04-10

**Authors:** Mingqi Zhou, Jordan B. Callaham, Matthew Reyes, Michael Stasiak, Alberto Riva, Agata K. Zupanska, Mike A. Dixon, Anna-Lisa Paul, Robert J. Ferl

**Affiliations:** ^1^Department of Horticultural Sciences, Program in Plant Molecular and Cellular Biology, University of FloridaGainesville, FL, USA; ^2^Exploration Solutions, Inc.Moffett Field, CA, USA; ^3^School of Environmental Sciences, University of GuelphGuelph, ON, Canada; ^4^Interdisciplinary Center for Biotechnology Research, University of FloridaGainesville, FL, USA

**Keywords:** *Arabidopsis thaliana*, microarray, low atmospheric pressure, hypobaria, hypoxia

## Abstract

Controlled hypobaria presents biology with an environment that is never encountered in terrestrial ecology, yet the apparent components of hypobaria are stresses typical of terrestrial ecosystems. High altitude, for example, presents terrestrial hypobaria always with hypoxia as a component stress, since the relative partial pressure of O_2_ is constant in the atmosphere. Laboratory-controlled hypobaria, however, allows the dissection of pressure effects away from the effects typically associated with altitude, in particular hypoxia, as the partial pressure of O_2_ can be varied. In this study, whole transcriptomes of plants grown in ambient (97 kPa/pO_2_ = 21 kPa) atmospheric conditions were compared to those of plants transferred to five different atmospheres of varying pressure and oxygen composition for 24 h: 50 kPa/pO_2_ = 10 kPa, 25 kPa/pO_2_ = 5 kPa, 50 kPa/pO_2_ = 21 kPa, 25 kPa/pO_2_ = 21 kPa, or 97 kPa/pO_2_ = 5 kPa. The plants exposed to these environments were 10 day old Arabidopsis seedlings grown vertically on hydrated nutrient plates. In addition, 5 day old plants were also exposed for 24 h to the 50 kPa and ambient environments to evaluate age-dependent responses. The gene expression profiles from roots and shoots showed that the hypobaric response contained more complex gene regulation than simple hypoxia, and that adding back oxygen to normoxic conditions did not completely alleviate gene expression changes in hypobaric responses.

## Introduction

On the Earth, the total atmospheric gas pressure of sea level is near 101 kPa with partial pressure of oxygen at 21 kPa. As elevation increases, the atmospheric pressure decreases until 0 kPa is reached at an altitude of about 30,000 m. In the first 5,000 m or so of elevation, a variety of biomes can be found populated with organisms adapted to the environmental factors associated with a climb in altitude, particularly the reduction of both temperature and available oxygen as the air pressure thins (Paul and Ferl, 2006). These attendant features of natural low pressure environments on Earth limit the range of life, and thus hypobaric conditions below about 40 kPa represent a novel environment—creating an opportunity to examine that is outside the evolutionary experience of plants. In the laboratory, hypobaric environments can be created that reduce the pressure of the atmosphere while maintaining amenable temperature, humidity, and even balance of gasses. In addition, as long as temperatures are maintained above freezing, and as long as there is sufficient water available to support the elevated transpiration brought on by the reduced atmospheric pressure, higher plants appear to physiologically adapt quite well to hypobaric environments, although the specific response of plants to hypobaric atmospheres varies greatly depending on the composition of the atmosphere, and even the plant species (Andre and Massimino, 1992; Corey et al., 2002; Goto et al., 2002; He et al., 2003; Paul et al., 2004). Depending on the partial pressures of oxygen, CO_2_, and volatiles such as ethylene, plant growth and development will typically be stressed in hypobaria to varying degrees of severity (Rule and Staby, 1981; Musgrave et al., 1988; Paul et al., 2004; He et al., 2007). However, the effects of hypobaric atmospheres on plant physiology are complex and not always detrimental. Enhanced photosynthesis has been observed in *Arabidopsis thaliana* exposed to moderate hypobaria (Richards et al., 2006) and in lettuce at moderately reduced partial pressure of oxygen (Corey et al., 1997). It has also been reported that plants grown in 25 or 30 kPa with even lower partial pressures of oxygen (2–6% O_2_) actually show higher gas exchange efficiency, higher bioactive component content, as well as enhanced morphological features, such as protected ultrastructure of mitochondria and chloroplasts compared with 101 kPa with same level of hypoxia (He et al., 2007; Tang et al., 2015). These studies suggest that hypobaric, hypoxic environments can induce adaptive measures in plants that contribute to protection from hypoxic injury more effectively than plants in an equally hypoxic environment at normal atmospheric pressure.

These complex metabolic responses demonstrate that plants are able to cope with hypobaric stress. Understanding the mechanisms behind this physiological adaptation is relevant to terrestrial crop breeding, particularly in the effort to expand croplands into marginal terrain and environments, and also to orbital and extraterrestrial controlled agriculture, where hypobaric environments may reflect a favorable engineering choice for plant growth habitats (Corey et al., 2002; Paul and Ferl, 2006; Wheeler, 2010). Although, the literature exploring physiological responses to hypobaric environments is growing, studies of the underlying changes in gene expression that contribute to these responses are limited.

Is hypobaria a simple combination of “familiar” terrestrial stresses? Primary among these component stresses might be the hypoxia resulting from the overall reduction of oxygen along with the balance of other gasses, as well as water stress due to the accelerated flux through stomata that accompanies the lowered air pressure (Iwabuchi and Kurata, 2003; Paul et al., 2004; Richards et al., 2006). In early transcriptome analyses of plants in hypobaric environments, genes encoding hallmarks of both hypoxic stress and drought stress were highly induced (Paul et al., 2004). The reduced partial pressure of oxygen (hypoxia) in hypobaric conditions is a major contributor to plant stress in these environments (Daunicht and Brinkjans, 1992; Ferl et al., 2002). Hypoxia physiologically inhibits respiration and oxidative phosphorylation leading to an energy deficit in plant cells (Drew, 1997; Mustroph et al., 2010). Transcriptomes from *Arabidopsis* in response to either a hypobaric environment of 10 kPa, or a hypoxic environment of 2% oxygen at 101 kPa, shared a large number of differentially expressed genes, indicating the similarity between hypobaria and hypoxia. However, many differentially expressed genes were unique to each treatment, suggesting that hypobaria is not equivalent to hypoxia (Paul et al., 2004). One grouping of genes that were differentially expressed in the hypobaric transcriptome were those typically associated with desiccation and abscisic acid signaling-related processes, even though the plants were fully hydrated, and showed no dehydration-associated phenotypes (Paul et al., 2004). These initial findings indicated plants potentially use water movement through stomata to gauge desiccation stress, and that plants utilize diverse sensing pathways to develop strategies for coping with the combination of a reduction in oxygen and an increase in evapotranspiration that is imposed by hypobaric environments. However, in these initial experiments, it was not possible to fully isolate the hypoxic effects from hypobaria.

The data presented here specifically endeavor to separate the effects of hypoxia and water stress from any other potential effects of hypobaria on plant biology, and to ask if there are specific responses that solely are driven by atmospheric pressure, and then to ask if those responses are all associated with water stress. The transcriptional profiles of *Arabidopsis* growing in atmospheric pressures of 50 or 25 kPa with supplemental oxygen were compared with straight hypobaria, in order to evaluate the consequence of removing the hypoxic stress component from the hypobaric environment. The genes still altered in low pressure with supplemental oxygen were then used to identify pathways associated with pure hypobaria.

## Materials and methods

### Chambers and facilities for low pressure treatments

**Figure 1:**
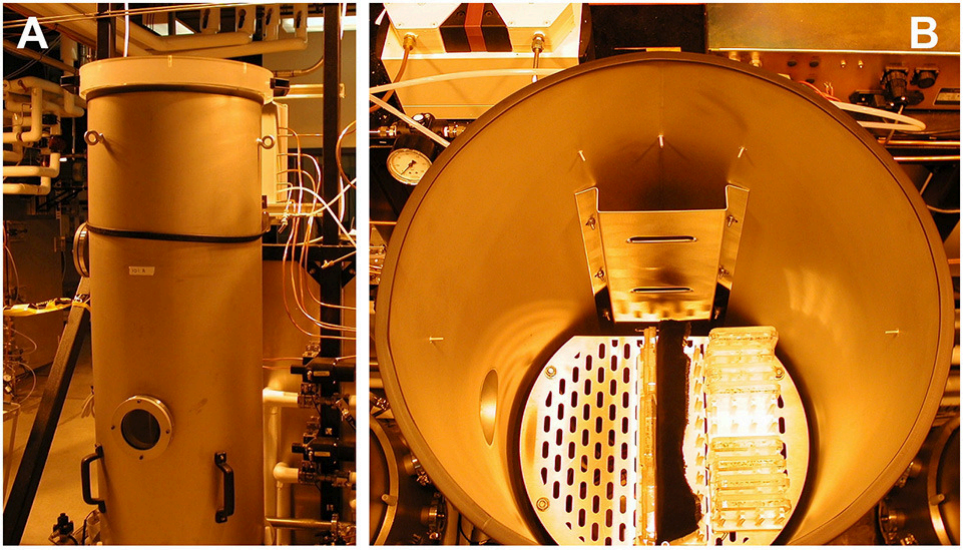
**Use of Low pressure growth chambers (LPGC). (A)** LPGCs were employed for hypobaric and hypoxic treatments. Individually, each LPGC chamber had controlled and monitored temperature, air pressure, gas regulation, as well as monitored relative humidity and vapor pressure density. The internal size of the chamber is 0.45 × 1.6 m (Diameter × Height) and the volume is 245 l. **(B)** Plates containing MS media were vertically orientated inside the LPGC. Plants grown in plates were placed in a constant light condition. LPGC were monitored on 5 min intervals with controlled temperature at 23°C ± 1°C, carbon dioxide of 0.05 kPa and humidity at or above 95%. The total pressure and oxygen partial pressure were set up as needed.

The Low Pressure Growth Chambers (LPGC) are components of the Controlled Environment Systems Research Facility (CESRF at University of Guelph, Ontario, Canada). Temperature, air pressure, gas composition and humidity were controlled and monitored in the LPGC by the second, and data from the LPGC were collected for reporting every 5 min. Control pressure was set at 97 kPa, which is the ambient pressure at an altitude of 1,100 feet (Guelph, Ontario). The LPGC provided a constant light condition (70–80 μmol m^−2^ s^−1^) with strictly monitored, controlled temperature at 22° to 24°C; the humidity was maintained inside the Petri plates at 95% or above (Figures 1A,B).

### Plant materials and growth condition

*A. thaliana* ecotype Wassilewskija (WS) was grown as previously described (Paul et al., 2001). In brief, seeds were surfaced sterilized and then planted on vertically orientated plates containing 0.5 × MS media (2.2 g of MS basal salts (Sigma, St. Louis), 5 g of Sucrose, 0.5 g of MES, and 1 mL of 1,000 × Gamborg vitamins (Sigma) per liter at pH 5.75), 0.45% Phytagel, and 2.5 ppm benomyl. Plants were grown for either 10 or 5 days in an ambient pressure growth chamber at CESRF with 24-h light, 22 to 24°C, 97 kPa prior to low pressure treatments.

### Variations on the pressure and oxygen supplement

For atmospheric treatments, 10 d plants were transferred to LPGCs and exposed to the following six treatments for 24 h: (1) 97 kPa, (2) 50 kPa, (3) 25 kPa, (4) 50 kPa with oxygen supplemented to a partial pressure of 21 kPa (defined as 50 kPa/NormOx), (5) 25 kPa with oxygen supplement to a partial pressure of 21 kPa (defined as 25 kPa/NormOx), and (6) 97 kPa with reduced oxygen to a partial pressure of 5 kPa (defined as 97 kPa/HypOx). At the same time, 5 d plants were transferred to LPGCs and exposed to 97, 50, and 50 kPa with oxygen supplemented to a partial pressure of 21 kPa (50 kPa/NormOx) for 24 h. The carbon dioxide was held constant at a partial pressure of 0.05 kPa in all treatments. Nitrogen was used as a balance of remaining gas for oxygen treatments. The light, temperature and humidity remained the same as mentioned above. Each atmospheric treatment was replicated in three different chambers, and each chamber held 10 individual plates comprised of 12 plants each.

### Plant harvest, RNA extraction, and quantitative tests

At the completion of each atmospheric treatment, plants were harvested from media surface directly to RNAlater (Ambion). For each treatment, there were three chambers each containing10 plates of plants in total. Approximately 12 plants from each plate were harvested to a separate tube and were immediately stored as previously described (Paul and Ferl, 2011). One tube was selected from each LPGC replicate, for a total of three tubes per treatment group. Total RNA was extracted using the Qiagen RNAeasy kit and genomic DNA was removed using RNase-free DNase. RNA samples quantity and quantity were assessed using the BioSpectrometer (Eppendorf) and Agilent 2100 Bioanalyzer (Agilent Technologies, Inc.).

### Microarray experiments

The 100 ng of total RNA were used for reverse transcription and preparation of biotin-labeled cRNA using the 3′ IVT plus Kit (Affymetrix). The Affymetrix GeneChip® Arabidopsis ATH1 Genome Arrays were hybridized with 12.5 μg purified and fragmented cRNA products for 16 h at 45°C. Arrays were washed using the Washing Procedure FS450_0004 and scanned with an Affymetrix GeneChip Scanner 3,000 7G. Primary data analysis was performed using the MAS5 algorithm within the Affymetrix Expression Console software. Array experiments were carried out at the Microarray Core of Interdisciplinary Center for Biotechnology Research, University of Florida. Array data are deposited in the Gene Expression Omnibus database with the accession number of GSE87869.

### Microarray data analysis

In total, 24 Affymetrix ATH1 arrays for roots and 18 for shoots were used in these analyses. Table 1 lists the number of arrays for each treatment. Only one array was available for analysis of 25 kPa/NormOx in roots due to technical problems in the array hybridization. The array data were normalized using the Robust Multi-chip Average (RMA) method and data quality was assessed using arrayQualityMetrics package for R/Bioconductor pipeline (Ritchie et al., 2015) and various QC charts (Density & Intensity plot, NUSE, RLE, and RNA Degradation Plot). Differential analysis between arrays was performed using the LIMMA package for R (Ritchie et al., 2015). Genes encoding mitochondrial or plastid transcripts were removed from the dataset. In our dataset, correction of *p* value led to failure of recognizing any genes in differential comparison of 50 vs. 97 kPa, 50 kPa/NormOx vs. 97 kPa, and 25 kPa/NormOx vs. 97 kPa in 10 d roots and shoots, and could only isolated very few genes in 25 vs. 97 kPa, and 97 kPa/HypOx vs. 97 kPa. To avoid missing true effects, we used *p* < 0.01 without correction for identification of differentially expressed genes. Although, adopting *p* < 0.01 without correction will increase the false positives, the alternative is the loss of all differentially expressed genes in selected comparisons of hypobaric response. Hierarchical clustering analysis was performed according to Kendall tau distance of Log2 fold-change of genes in the dataset and heat maps were graphed using GENE-E (http://www.broadinstitute.org/cancer/software/GENE-E/). Biological process ontology analyses were carried out using AgriGO (http://bioinfo.cau.edu.cn/agriGO/index.php) (Du et al., 2010). GO terms with *p* < 0.01 were listed in Tables S1–S3. Pathway enrichment was annotated with DAVID6.8 online tools (https://david.ncifcrf.gov/) according to Kyoto Encyclopedia of Genes and Genomes (KEGG) database (http://www.genome.jp/kegg/tool/map_pathway1.html) (Huang et al., 2009).

**Table 1:** **Replicates of array at each treatment**.

**Treatments**	**97 kPa**	**50 kPa**	**25 kPa**	**50 kPa/NormOx**	**25 kPa/NormOx**	**97 kPa/HypOx**
	**Normal pressure**	**Low atmospheric pressure Total pressures (pTOT)**		**Hypobaria with normal oxygen composition (normoxia)**		**Hypoxia with normal pressure**
	Ambient at Guelph, ON	pO_2_ = 10 kPa	pO_2_ = 5 kPa	pTOT 50 kPa −pO_2_ = 21 kPa	pTOT 25 kPa −pO_2_ = 21 kPa	pTOT 97 kPa −pO_2_ = 5 kPa
10 day old Roots	3	3	3	2	1	3
10 day old Shoots	3	3	3	4	3	2
5 day old Roots	3	3	−	3	−	−

### Taqman quantitative RT-PCR

Quantitative RT-PCR was employed to quantify the expression levels of genes selected from the microarrays data. RNA from the same 10 d roots samples used for microarray analysis as well as additional experimental replications were subjected to this confirmation. The Applied Biosystems Prism 7700 Sequence Detection System was used for the qRT-PCR analysis (Paul et al., 2004). The fluorescently tagged probes and paired primers flanking a 60–100 bp section of the gene of interest are listed in Table 2. The gene expression level was normalized by relating the Taqman results to a standard curve. UBQ11 (AT4G05050) was used as the internal control. Three replicates for each sample were used.

**Table 2:** **Primers and probes used for Taqman qRT-PCR**.

**Gene**	**Name**	**Sequence (5′-3′)**
AHB1	AHB1-Forward	GGTGGCCAAGTATGCATTGTT
(AT2G16060)	AHB1-Probe	AGACGATAAAGGAGGCAGTGCCGGA
	AHB1-Reverse	CCCCAAGCCACCTTCATCT
PDC1	PDC1-Forward	GCTCTGTTGGTTACTCGCTTCTC
(AT4G33070)	PDC1-Probe	TCAAGAAAGAAAAAGCCATCGTTGTGCAA
	PDC1-Reverse	TGGCCACAGTGATACGATCAG
UBQ11	UBQ11-Forward	AACTTGAGGACGGCAGAACTTT
(AT4G05050)	UBQ11-Probe	CAGAAGGAGTCTACGCTTCATTTGGTCTTGC
	UBQ11-Reverse	GTGATGGTCTTTCCGGTCAAA

## Results and discussion

### Plant phenotypes in low atmospheric pressure environment

**Figure 2:**
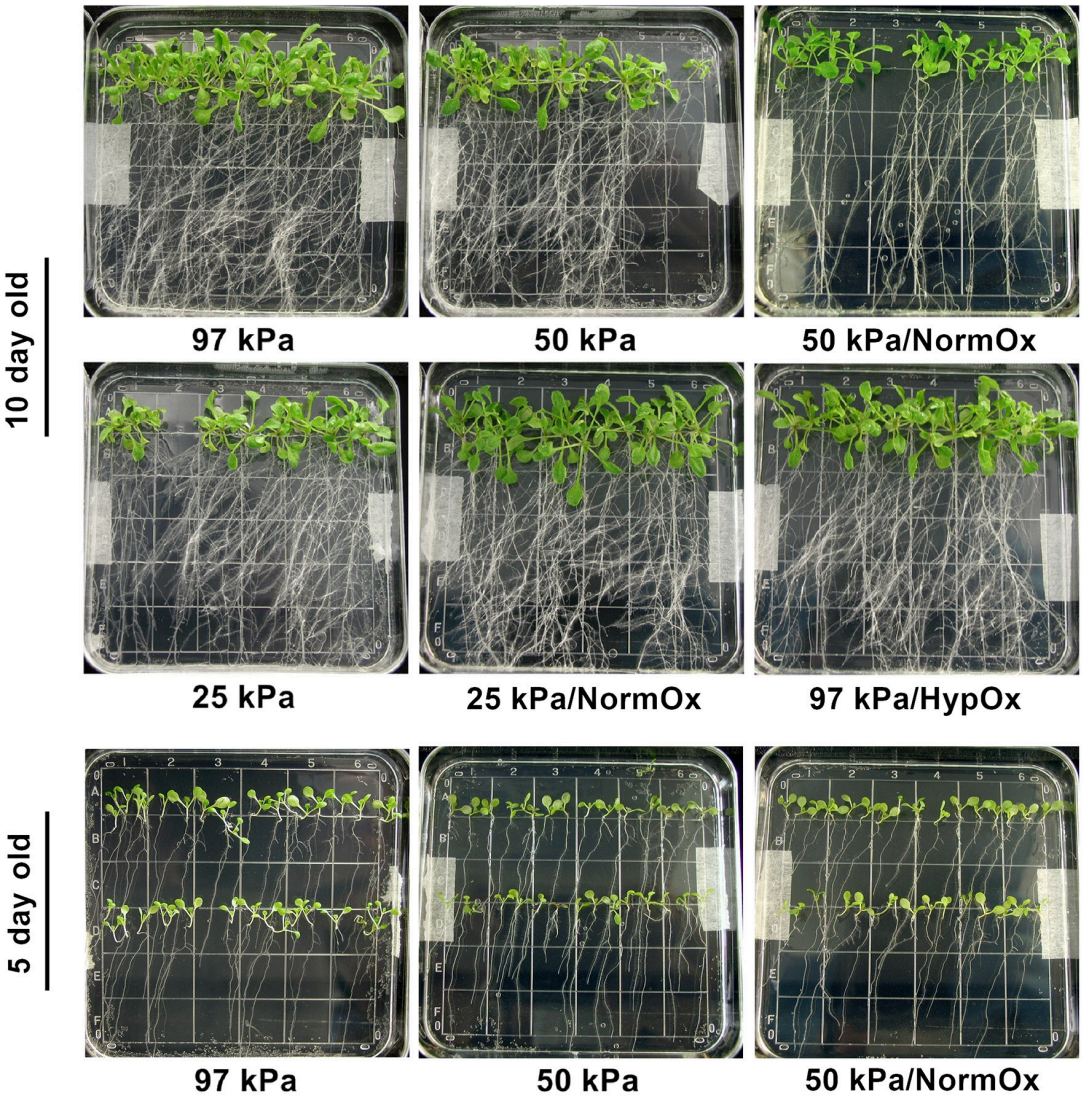
**Phenotype of plants treated with 24 h of hypobaric or hypoxic stress as indicated**. Photos were taken right after the stress application. 10 d seedlings undergoing 97 kPa, 50 kPa, 50 kPa/NormOx (50 kPa/pO_2_ = 21 kPa), 25 kPa, 25 kPa/NormOx (25 kPa/pO_2_ = 21 kPa), and 97 kPa/HypOx (97 kPa/pO_2_ = 5 kPa), as well as 5 d seedlings treated with 97 kPa, 50 kPa and 50 kPa/NormOx were shown. None of 5 d or 10 d plants under these treatments showed desiccation-associated phenotype.

Other that minor changes associated with 24 h of growth, the phenotypes of the plants after the hypobaric conditions used in these experiments did not change (data not shown). As can be seen in the post-treatment photographs of Figure 2, there was no evidence of desiccation-associated phenotypes, such as leaf wilting, observed in either the 10 day old (10 d) or 5 day old (5 d) seedlings across the hypobaric and hypoxic treatments (Figure 2). This observation is consistent with our previous study in 10 kPa and 2% O_2_ conditions (Paul et al., 2004). The high relative humidity and continuous water supply provided by the micro-climate of the Petri plate growing system likely removes any outward expression of actual dehydration. The solid nutrient media in the petri plant growing system is sufficient to grow Arabidopsis for 24 h in severe hypobaria without exhausting the water supply within the media. Although, water is moving more rapidly through the stomata, this phenomenon does not cause a visible (e.g., wilted) phenotype.

### The impact of supplemental oxygen on transcriptional responses to 50 kPa atmospheres

**Figure 3:**
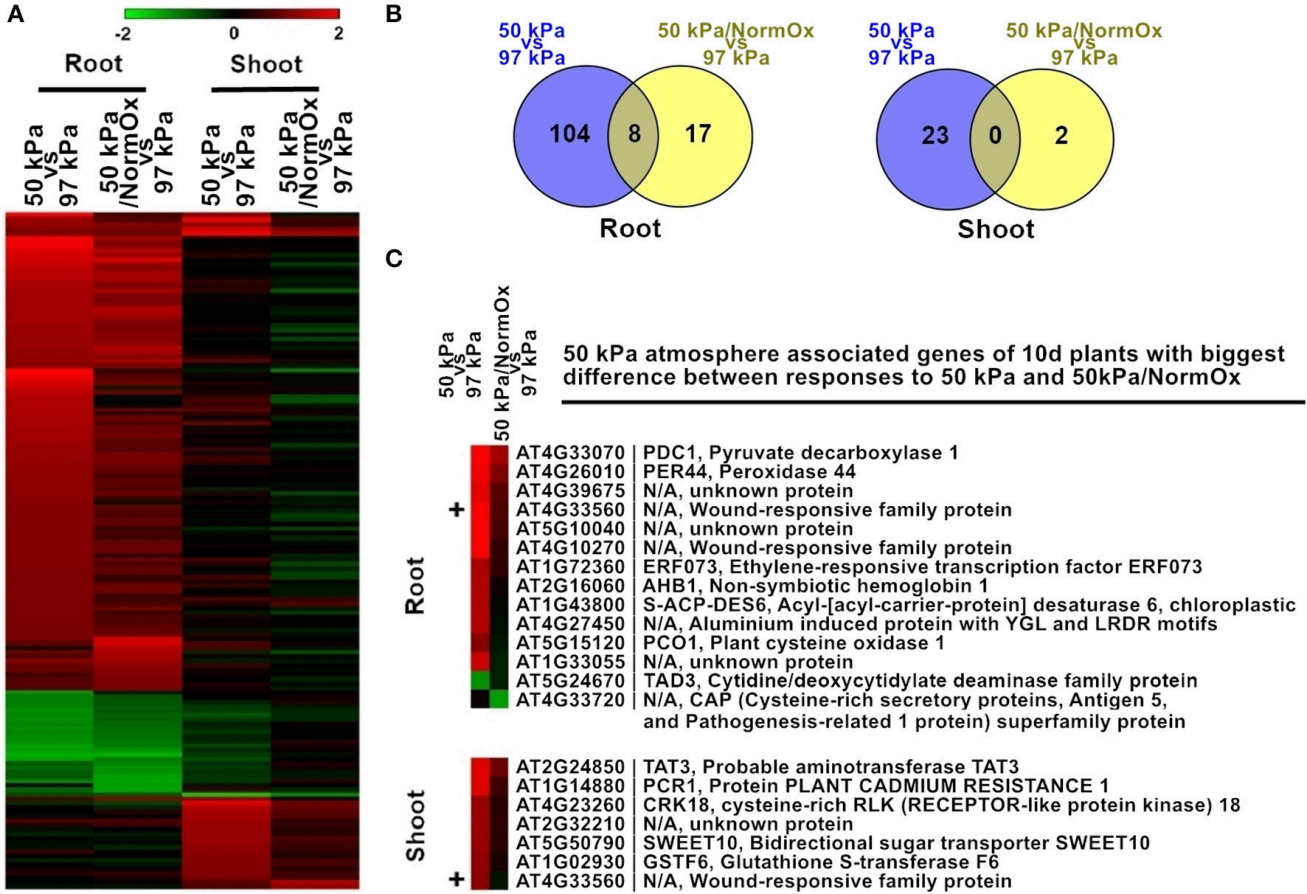
**Differentially expressed genes in response to 50 kPa and 50 kPa/NormOx in roots and shoots of 10 d plants. (A)** Heat map of 151 differentially expressed genes with statistical significance (*p* < 0.01) by at least 2-fold in at least one of 50 kPa and 50 kPa/NormOx (50 kPa/pO_2_ = 21 kPa) responses in roots or shoots. Filled colors represent Log2 fold-change. **(B)** Venn diagram showing the overlap of significantly changed genes (Log2 fold-change >1, *p* < 0.01) between responses to 50 kPa and 50 kPa/NormOx in roots and shoots. **(C)** 50 kPa atmosphere associated genes of 10 d plants with biggest difference in expression level between responses to 50 kPa and 50 kPa/NormOx. The genes with difference of expression level more than 1.8-fold in roots and shoots were listed. The “+” represents the gene shared by the lists of roots and shoots.

Two treatments were used to evaluate the contribution of hypoxia to the hypobaric transcriptome of *Arabidopsis*: exposing seedlings to 50 kPa, and to 50 kPa supplemented with oxygen to a normal (21 kPa) level (50 kPa/NormOx) (Figure 3). Genes with significant (*p* < 0.01) differential expression by at least 2-fold based on 97 kPa control were defined as differentially expressed genes. There were 151 differentially expressed genes identified in at least one of these two conditions in roots or shoots, which were defined as 50 kPa atmosphere associated genes of 10 d plants (Figure 3A). Most of these genes exhibited different expression patterns in roots and shoots, demonstrating the tissue-specific response to hypobaria. Among them, 112 genes in 50 kPa and 25 genes in 50 kPa/NormOx were significantly changed in roots, while 23 genes in 50 kPa and 2 genes in 50 kPa/NormOx were in shoots (Figure 3B), suggesting that roots could be more sensitive and shoots might possess better adaptation ability especially to 50 kPa/NormOx. According to expression patterns, these 151 genes were clustered and GO analysis was performed for each gene clade (Table S1). In general, most genes up- or down-regulated in 50 kPa were associated with abiotic stimulus and metabolic process in roots. One example for drought and cold responsive genes was Sucrose Synthase 1 (SUS1, AT5G20830) (Déjardin et al., 1999), which was significantly induced by 50 kPa treatment. When contrasted, shoots showed small overlap with roots and elevated a set of biotic stimulus regulated genes. Only one biotic gene, a thionin gene (AT1G72260) responsive to pathogens, was down-regulated in shoots. For 50 kPa/NormOx response, both roots and shoots involved metabolism associated genes but none of them overlapped. These demonstrated that different plant organs/tissues could generate diverse responses to hypobaria with or without hypoxia.

As revealed by the heat map pattern shown in Figure 3, some differentially expressed genes that were significantly changed at least in one condition had similar trends of behavior in 50 kPa and 50 kPa/NormOx. However, if selected using a stringent criteria of fold change as well as *p* value in each condition, few genes were overlapped between 50 kPa and 50 kPa/NormOx (Figures 3A,B). To further clarify the difference between plant responses to 50 kPa and 50 kPa/NormOx, 129 differentially expressed genes in roots and 25 genes in shoots were used to assess expression distinction. Surprisingly, there were only 14 genes in roots and 7 genes in shoots showing more than 1.8-fold in expression difference between responses to 50 kPa and 50 kPa/NormOx, and one of them, AT4G33560 that is a wounding responsive unknown gene, was shared (Figure 3C). These genes revealed the biggest difference between responses to 50 kPa and 50 kPa/NormOx. In roots, 12 genes including typical hypoxia responsive genes such as PDC1 (AT4G33070), AHB1 (AT2G16060), and PCO1 (AT5G15120) were highly expressed in 50 kPa but not 50 kPa/NormOx (Gibbs et al., 2011; Licausi et al., 2011a). Meanwhile, only one gene named TAD3 (AT5G24670) was repressed in 50 kPa but not 50 kPa/NormOx. It has been reported that the knockout of TAD3 was lethal and knockdown mutation of which led to reduced plant growth (Zhou et al., 2014), suggesting that the combined hypobaria might trigger growth repression pathways, which was consistent with the previously reported reduced plant growth in long-term exposure to 30 kPa/pO_2_ = 6 kPa compared with ambient control of 101 kPa/pO_2_ = 21 kPa (Tang et al., 2015). The unknown gene AT4G33720 belonging to the CAP (Cysteine-rich secretory protein) superfamily was reported to be regulated by DREB2A, a key transcription factor in drought response (Sakuma et al., 2006). Seven of the genes were up-regulated in shoots at 50 kPa but not 50 kPa/NormOx, and five of which were associated with signal transduction (Figure 3C).

The pressure of 50 kPa, half of the barometric pressure at sea level (around 101 kPa), can be considered as a boundary of moderate and severe hypobaria, as the natural terrestrial limit of atmosphere for higher plants is no less than 50 kPa (Paul et al., 2004; Richards et al., 2006). The oxygen partial pressure of 10.3 kPa in 50 kPa is a mild hypoxic stress, which causes no obvious change in vegetative growth of Arabidopsis plants in soil (Ramonell et al., 2001). In the present work, comparison between 50 kPa and 50 kPa/NormOx distinguishes the responses to hypobaria with and without hypoxia in moderate low pressure. In summary, even though 50 kPa involved a weak hypoxic stress, the transcriptome of 50 kPa and 50 kPa/NormOx treatments did not show a dramatic difference with respect to the hypobaric component of the environment. In addition, plant responses to hypobaria with or without hypoxia were shown to be regulated in a tissue-specific manner.

### The impact of developmental age on the transcriptional response to 50 kPa atmospheres

**Figure 4:**
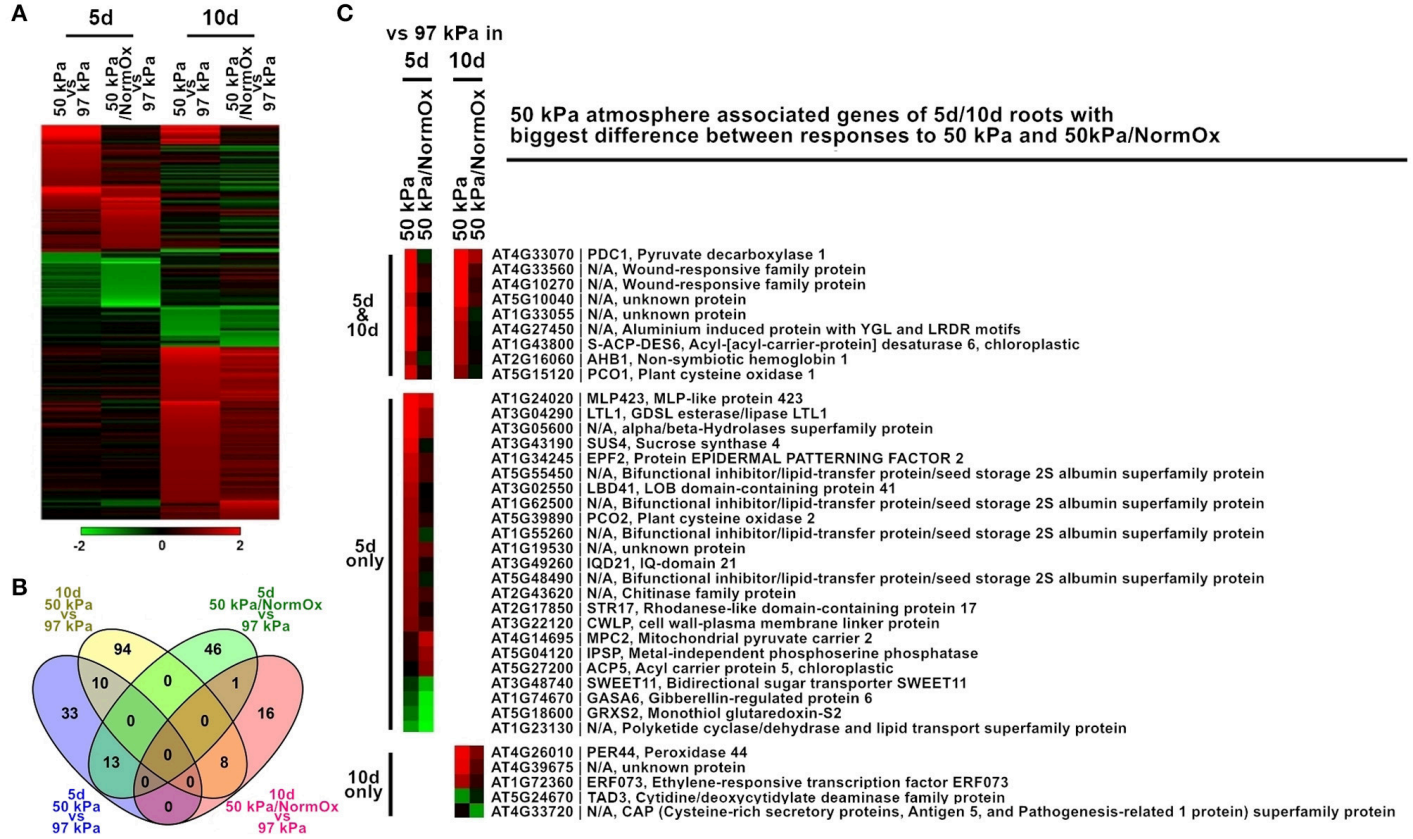
**Differentially expressed genes in response to 50 kPa and 50 kPa/NormOx in roots of 10 d and 5 d plants. (A)** Heat map of 221 differentially expressed genes with statistical significance (*p* < 0.01) by at least 2-fold in at least one of responses to 50 kPa and 50 kPa/NormOx (50 kPa/pO_2_ = 21 kPa) in roots of 10 d or 5 d plants. Filled colors represent Log2 fold-change. **(B)** Venn diagram showing the overlap of significantly changed genes (Log2 fold-change >1, *p* < 0.01) between responses to 50 kPa and 50 kPa/NormOx in roots of 10 d and 5 d plants. **(C)** 50 kPa atmosphere associated genes of 5 d/10 d roots with biggest difference in expression level between responses to 50 kPa and 50 kPa/NormOx. The genes with difference more than 1.8-fold were listed.

The developmental age of *Arabidopsis* had a substantial effect on the profile of differentially expressed genes in response to the 50 kPa and 50 kPa/NormOx atmospheres. The root transcriptomes of 5 d *Arabidopsis* seedlings revealed exhibited notably different expression patterns than the transcriptomes of the 10 d roots in the same environments (Figure 4). There were 221 differentially expressed genes in at least one of these two conditions in roots of 5 or 10 d plants, which were subsequently defined as 50 kPa atmosphere associated genes of 5/10 d roots (Figure 4A). Among them, 103 genes of 5 d roots and 129 genes of 10 d roots were identified. Only 10 genes were coordinately expressed in 5 d and 10 d roots in 50 kPa, and just a single gene in the 50 kPa/NormOx treatments (Figure 4B). The clustered differentially expressed genes were listed in Table S2 and GO terms of each clade were shown. In response to 50 kPa, 5 d plants had more expressed genes associated with lipid metabolism changes while 10 d plants showed more abiotic stress response. They shared typical hypoxia responsive genes including PDC1, AHB1, and PCO1. For response to 50 kPa/NormOx, only one gene, bidirectional sugar transporter SWEET11 (AT3G48740), was repressed in both 5 and 10 d plants. Other differentially expressed genes for 50 kPa/NormOx were mostly associated with metabolic process but none of them were shared by the two different ages. To further conduct the comparison of age-dependent responses to 50 kPa and 50 kPa/NormOx, the 103, and 129 of 50 kPa atmosphere associated genes identified in 5 or 10 d roots were studied to show expression differences between responses to 50 kPa and 50 kPa/NormOx. Among these, 37 genes exhibited more than 1.8-fold in expression difference between responses to 50 kPa and 50 kPa/NormOx, including 23 genes only in 5 d plants, 5 genes only in 10 d plants and 9 genes in both ages (Figure 4C). Hypoxia and wound responsive genes shared by 5 and 10 d plants composed the age-independent difference between responses to 50 kPa and 50 kPa/NormOx. The 5 d roots used more lipid, sugar and water metabolism related genes in response to 50 kPa but not 50 kPa/NormOx. The stomatal density and development controlling protein Epidermal Patterning Factor 2 (EPF2) (AT1G34245) that would increase water use-efficiency (Engineer et al., 2014; Franks et al., 2015) and cell wall-plasma membrane linker protein (CWLP) (AT3G22120) that could contribute to water content protection (Stein et al., 2011) were only up-regulated in response to 50 kPa. Interestingly, most of these genes were not responsive to either 50 kPa or 50 kPa/NormOx in 10 d plants, suggesting that hypobaria triggers different responsive pathways in developmental ages of plants.

### Comparison of transcriptional responses to hypobaria and hypoxia with respect to 25 kPa atmospheres

**Figure 5:**
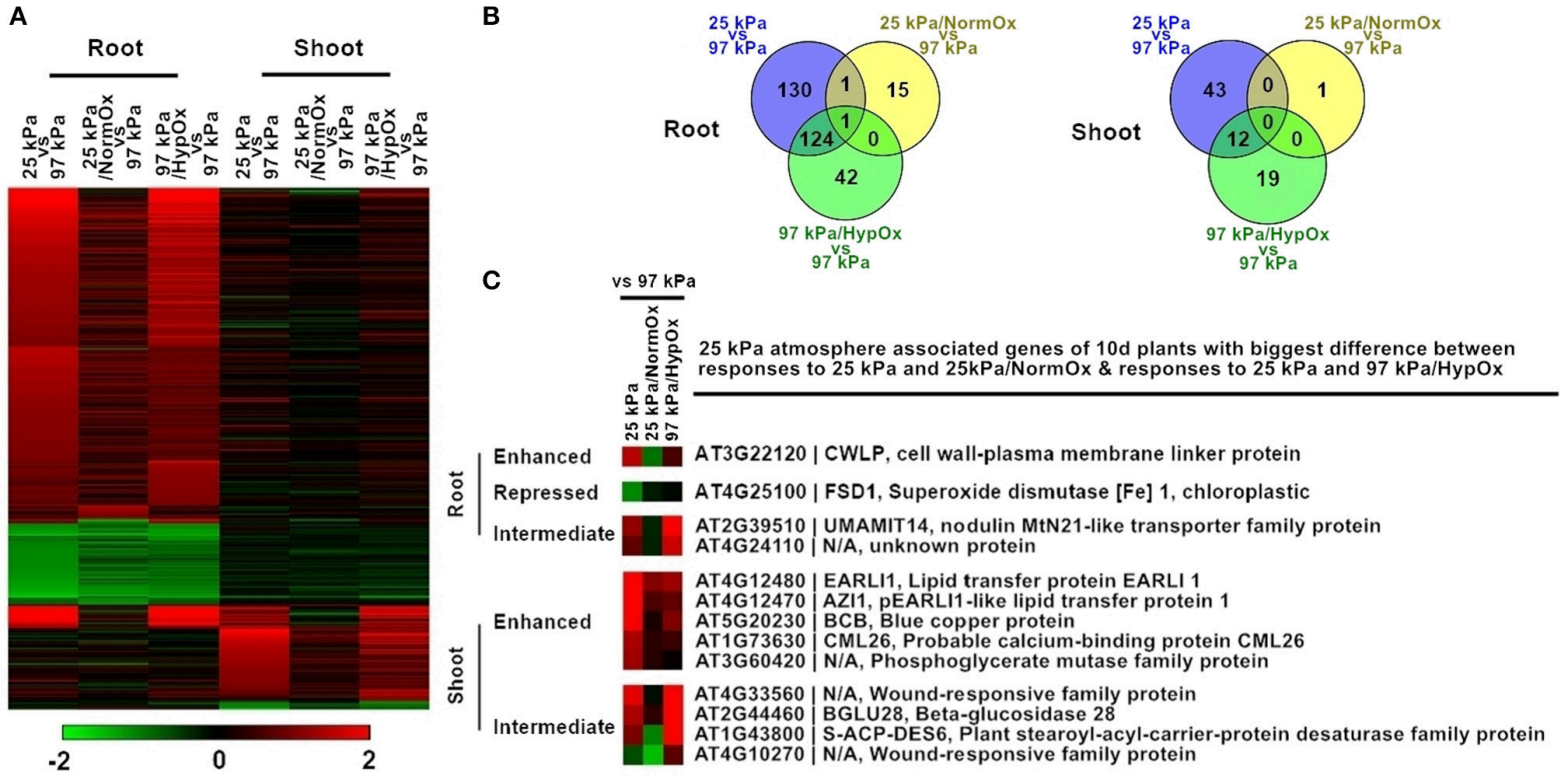
**Differentially expressed genes in response to 25 kPa, 25 kPa/NormOx, and 97 kPa/HypOx in roots and shoots of 10 d plants. (A)** Heat map of 372 differentially expressed genes with statistical significance (*p* < 0.01) by at least 2-fold in at least one of responses to 25 kPa, 25 kPa/NormOx (25 kPa/pO_2_ = 21 kPa), and 97 kPa/HypOx (97 kPa/pO_2_ = 5 kPa) in roots or shoots. Filled colors represent Log2 fold-change. **(B)** Venn diagram showing the overlap of significantly changed genes (Log2 fold-change >1, *p* < 0.01) between response to 25 kPa, 25 kPa/NormOx, and 97 kPa/HypOx in roots and shoots. **(C)** 25 kPa atmosphere associated genes of 10 d plants with biggest difference in expression level between responses to 25 kPa and 25 kPa/NormOx as well as responses to 25 kPa and 97 kPa/HypOx. The genes with difference more than 1.8-fold in roots and shoots were listed. These genes were categorized as “enhanced,” “repressed,” and “Intermediate” according to expression difference.

The more severe hypobaric environment of 25 kPa was used to further dissect the contribution of oxygen in the transcriptional response of 10 d *Arabidopsis* to reduced atmospheric pressure (Figure 5). The partial pressure of oxygen at 25 kPa is 5 kPa, an oxygen concentration which is known to confer a strong hypoxic stress response in plants (Van Dongen et al., 2009; Mustroph et al., 2010). To better dissect hypobaric stress in 25 kPa, two additional conditions were utilized: total gas pressure in 25 kPa with a partial pressure of supplemental oxygen in 21 kPa (defined as 25 kPa/NormOx) and hypoxia containing total gas pressure in 97 kPa with a partial pressure of oxygen in 5 kPa (defined as 97 kPa/HypOx). Array analysis showed that there were 372 differentially expressed genes in at least one condition of 25 kPa, 25 kPa/NormOx, or 97 kPa/HypOx in roots or shoots, which were therefore defined as 25 kPa atmosphere associated genes of 10 d plants (Figure 5A). These genes were clustered and GO terms of each group were listed in Table S3. Overall, the difference between responses to 25 kPa and 25 kPa/NormOx was bigger than that of 25 kPa and 97 kPa/HypOx, indicating that hypoxic stress played a large role in severe low atmospheric pressure. Similar to 50 kPa, most of these 372 genes exhibited different expression patterns in roots and shoots. In roots, 256, 17, and 167 genes were significantly changed in 25 kPa, 25 kPa/NormOx, and 97 kPa/HypOx, respectively (Figure 5B). Only one gene, LBD41 (LOB domain-containing protein 41) (AT3G02550), was significantly changed in three responses, whereas it was upregulated in 25 kPa and 97 kPa/HypOx but downregulated by 25 kPa/NormOx. LBD41 was reported as a hypoxia and plant-parasitic nematodes responsive gene in *Arabidopsis* (Fuller et al., 2007; Licausi et al., 2011b). In response to 25 kPa, photosynthesis and energy metabolism associated genes were induced in addition to the stress responsive genes shared by response to 97 kPa/HypOx (Table S3). In shoots, 55, 1, and 31 genes were significantly changed in 25 kPa, 25 kPa/NormOx, and 97 kPa/HypOx, respectively (Figure 5B). Genes upregulated by 25 kPa were also largely involved in abiotic stress and defense responses, such as the desiccation associated gene Allene Oxide Cyclase 1 (AT3G25760). Shoots may be less sensitive to the reduction of atmospheric pressure as long as oxygen level was normal, since 25 kPa/NormOx with normoxia hardly activate gene expression changes in shoots. The one gene induced by 25 kPa/NormOx in shoots encoded a R2R3 factor MYB family protein, MYB44 (AT5G67300), which was identified as a positive regulator of salt, oxidative and innate immune responses as well as a negative regulator of ABA signaling and wounding response (Jaradat et al., 2013; Shim and Choi, 2013; Persak and Pitzschke, 2014). The absence of other genes showing altered expression with more than 2-fold in response to 25 kPa/NormOx may be due to the strength of transcriptional activation and/or the time point chosen for sampling of tissue. Plant transcriptional responses were not as sensitive to hypobaria without hypoxia in both moderate and severe low atmospheric pressure level, especially for shoots. For both roots and shoots, hypoxia responsive genes and a few of non-hypoxic associated genes in 25 kPa response were not responsive to 25 kPa/NormOx, which indicated that oxygen supplement restored hypoxia-caused changes as well as some other effects happened in hypobaria (25 kPa).

Similar to 50 kPa and 50 kPa/NormOx, difference of transcription pattern between responses to 25 kPa and 25 kPa/NormOx as well as 25 kPa and 97 kPa/HypOx must be clarified through direct comparison. Among 372 of 25 kPa atmosphere associated genes identified in Figure 5A, there were 271 genes in roots and 56 genes in shoots significantly changed in at least one of 25 kPa and 25 kPa/NormOx, and 298 genes in roots and 74 genes in shoots significantly changed in at least one of 25 kPa or 97 kPa/HypOx. In total, 93 genes between 25 kPa and 25 kPa/NormOx and 5 genes between 25 kPa and 97 kPa/HypOx showed more than 1.8-fold in expression change in roots, and 44 genes between 25 kPa and 25 kPa/NormOx and 11 genes between 25 kPa and 97 kPa/HypOx detected with same criteria in shoots (Table S3). These genes represented the differential response to “hypobaria together with hypoxia” (25 kPa) and “hypobaria without normoxia” (25 kPa/NormOx) or “hypoxia” (97 kPa/HypOx). For severe low atmospheric pressure level (25 kPa) in which hypoxic stress was strong, the difference between responses to 25 kPa and 97 kPa/HypOx were quite small in both roots and shoots. Majority of genes differentially expressed between responses to 25 kPa and 25 kPa/NormOx did not show difference between responses to 25 kPa and 97 kPa/HypOx. However, most of genes differentially expressed between 25 kPa and 97 kPa/HypOx were also significantly changed between 25 kPa and 25 kPa/NormOx, suggesting that a large portion of hypobaric responses were prompted by hypoxia, not total gas pressure, in this oxygen level. Nevertheless, 4 genes in roots and 9 genes in shoots simultaneously showed more than 1.8-fold in expression difference between responses to 25 kPa and 25 kPa/NormOx as well as responses to 25 kPa and 97 kPa/HypOx (Figure 5C). These 13 genes were categorized as “Enhanced” (with higher expression level in 25 kPa than that in 25 kPa/NormOx and 97 kPa/HypOx), “Repressed” (with lower expression level in 25 kPa than that in 25 kPa/NormOx and 97 kPa/HypOx) and “Intermediate” (with intermediate expression level in 25 kPa between that in 25 kPa/NormOx and 97 kPa/HypOx). For “Enhanced” group there was 1 gene in roots and 5 genes in shoots (Figure 5C). The “enhanced” root gene CWLP was also induced by 50 kPa in 5 d plants, which was involved in water and lipid metabolism (Stein et al., 2011). Two of the “enhanced” shoot genes also belonged to lipid transfer protein family. There was one gene in “Repressed” group in roots and none in shoots, and the root gene, FSD1 (AT4G25100), could be induced by oxidative stress (Vanhoudt et al., 2010). Meanwhile, 2 genes in roots and 4 genes in shoots were in “Intermediate” group and all of them showed high expression in response to 97 kPa/HypOx and low in response to 25 kPa/NormOx, suggesting the negative regulation of these genes by lower total gas pressure. It is likely that these differentially responsive genes between hypobaria and hypoxia may play a role in mediating plant tolerance to low oxygen environments. For instance, in identical oxygen deficit conditions, plants can show more robust growth in hypobaric environments than under full atmospheric pressure (He et al., 2007), which implies that exposure to the combined environment induces adaptive strategies to better cope with a reduced availability of oxygen.

### Pathway enrichment of differentially expressed genes with respect to 50 and 25 kPa atmospheres

After identification of differentially expressed genes, we sought to explore metabolic pathways involved in responses to 50 and 25 kPa atmospheres. The KEGG pathways of significant enrichment with Benjamini corrected *p* < 0.05 were found by the tool DAVID using differentially expressed genes in each treatment (Table 3). Interestingly, no significant pathway was observed in 50 kPa or 50 kPa/NormOx in either roots or shoots, which was unlike the results of GO analysis. The differentially expressed genes mapped in the GO terms cannot form significant enrichment of known pathways, indicating that plant may possess different strategies to cope with hypobaria compared with other common abiotic stresses. For 25 kPa associated conditions, genes of 25 kPa/NormOx did not show any significant pathway enrichment either. When hypoxia was included, pathway enrichment was observed for 25 kPa and 97 kPa/HypOx and the mapped genes were listed in Table S4. Although, there were much more 25 kPa responsive genes, fewer pathways were identified for genes of 25 kPa compared with 97 kPa/HypOx in both roots and shoots. There were 15.4% of differentially expressed genes of 25 kPa in roots mapped to “Metabolic pathways” and not a single specific pathway was detected. For genes of 97 kPa/HypOx, 5.4, 19.3, and 12.7% were mapped to “Glycolysis/Gluconeogenesis,” “Metabolic pathways” and “Biosynthesis of secondary metabolites” in roots. Altered glycolysis is one of the typical hypoxia related metabolic changes (Liu et al., 2005). In shoots, 7.3% of 25 kPa genes were mapped to “alpha-Linolenic acid metabolism.” The alpha-Linolenic acid level is related to salt, drought and hypoxia (Zhang et al., 2012; Klinkenberg et al., 2014). Compared with previously reported differentially expressed genes in 10 kPa (Paul et al., 2004), fewer drought responsive genes were identified in 50 kPa and 25 kPa. The absence of core drought responsive genes such as COR15A and RD29a could be due to the weaker severity of hypobaric stress. Here significantly enriched metabolic pathway suggested the moderate water-loss or desiccation response in 25 kPa. For 97 kPa/HypOx, 38.7, 12.9, 29, and 9.7% of responsive genes were mapped to “Metabolic pathways,” “Cysteine and methionine metabolism,” “Biosynthesis of secondary metabolites” and “alpha-Linolenic acid metabolism,” respectively. Overall, 97 kPa/HypOx caused some typical hypoxia related alterations, whereas the responses to hypobaria with or without hypoxia include a big proportion of unknown genes as well as genes that are of known functions but cannot be mapped to known pathways, suggesting the novelty of unique tools plants used to survive in environments that are out of evolutionary experience.

**Table 3:** **KEGG Pathway Enrichment of differentially expressed genes in 10 d plants (Benjamini corrected ***p*** < 0.05)**.

**Treatments**		**Differentially expressed genes**	**Pathways**	**Genes mapped to each pathway**	**Benjamini**
Root	50 kPa	112; e.g., AT4G26010, AT4G10270, AT4G33560, AT5G24670,	N/A	N/A	N/A
	50 kPa/NormOx	25; e.g., AT4G14630, AT1G78860, AT3G23190, AT5G47450,	N/A	N/A	N/A
Shoot	50 kPa	23; e.g., AT1G02930, AT2G24850, AT1G02850, AT4G35180,	N/A	N/A	N/A
	50 kPa/NormOx	2: AT5G18600, AT1G23130,	N/A	N/A	N/A
Root	25 kPa	256; e.g., AT5G15120, AT4G25110, AT3G58810, AT2G20630,	Metabolic pathways	39 (15.4%)	7.90E-04
	25 kPa/NormOx	17; e.g., AT2G07671, AT1G72440, AT5G03440, AT3G02550,	N/A	N/A	N/A
	97 kPa/HypOx	167; e.g., AT5G54960, AT1G77120,	Glycolysis/Gluconeogenesis	9 (5.4%)	2.20E-04
		AT1G17290, AT1G72360,	Metabolic pathways	32 (19.3%)	1.50E-04
			Biosynthesis of secondary metabolites	21 (12.7%)	4.30E-03
Shoot	25 kPa	55; e.g., AT3G25760, AT5G42650, AT4G15440, AT3G45140,	alpha-Linolenic acid metabolism	4 (7.3%)	9.40E-03
	25 kPa/NormOx	1: AT5G67300	N/A	N/A	N/A
	97 kPa/HypOx	31; e.g., AT1G75280, AT2G39310,	Metabolic pathways	12 (38.7%)	8.50E-03
		AT5G48880, AT2G31390,	Cysteine and methionine metabolism	4 (12.9%)	1.70E-02
			Biosynthesis of secondary metabolites	9 (29%)	2.00E-02
			alpha-Linolenic acid metabolism	3 (9.7%)	2.70E-02

### Adjustment of oxygen in space missions

Being able to mitigate the effects of atmospheres modified from “earth normal” is central to the exploration mission to take biology off planet, but understanding how plants dissect components of their environment to physiologically adapt to a stress is crucial to all plant cultivation scenarios. In the dissection of the hypobaria and hypoxic responses, the organ-specific and age-dependent transcription patterns suggest plants use adaptive strategies tied to the unique needs of a specific tissue, and that those needs change as the plant develops. Notably, the continuous light that matched the hardware in International Space Station (ISS) was used in our experimental process including plant germination, growth and all treatments. It was reported that light signal could affect hypoxic response especially in tissue-dependent gene induction (Van Veen et al., 2016). Low oxygen promotes accumulation of diverse metabolites in roots and shoots as well as in plants treated with different levels (5–10 kPa or ~ 0 kPa) or types (submergence, flooding or argon-gassed) of hypoxia (Miyashita and Good, 2008; Mustroph et al., 2014). Consistent with previous study of hypoxia response (Mustroph et al., 2014), 97 kPa/HypOx (97 kPa/pO_2_ = 5 kPa) highly induced ethanol metabolic genes including ADH1 (AT1G77120) and PDC2 (AT5G54960) as well as alanine associated gene ALAAT1 (AT1G17290) in roots but not shoots. However, in a recent study most of the previously defined core hypoxia genes (Mustroph et al., 2009) were induced in an organ-independent manner by flooding when plants were submerged in early stage of photoperiod (Van Veen et al., 2016), while in our present data 34 out of 51 core genes were associated with 25 kPa atmosphere and only 9 genes showed similar induction by 97 kPa/HypOx in roots and shoots (Table S3). These suggest that the growth condition with constant light started from germination might alter organ-specific regulation of hypoxia genes compared with other photoperiod conditions. Considering the facility limitation in spaceflight mission, it will be necessary to conduct respective studies on plant responses to oxygen changes in allusion to different light conditions of growth.

The oxygen supplement assays provided novel insight into plant transcriptomic response in the condition of low atmospheric pressure with adjusted partial pressures of O_2_, such as has been utilized in manned space missions in the past, and may be employed in the future. When oxygen is supplemented to both moderate and severe hypobaric conditions it minimizes transcriptional responses caused by low atmospheric pressure, with the implication that the overall stress load on the plant is reduced. In comparison between hypobaria without hypoxia in 50 and 25 kPa, 40 genes in roots and 3 genes in shoots were significantly changed in at least one of 50 and 25 kPa/NormOx conditions (Figure S1). The normoxic conditions with different total gas pressure resulted in similar transcription trends in these genes from both roots and shoots, which supported the conclusion from comparisons of 50 kPa vs. 50 kPa/NormOx and 25 kPa vs. 25 kPa/NormOx that plants were not so sensitive to hypobaria with nomoxia compared with hypobaria with hypoxia. However, while increasing oxygen concentrations in hypobaric environments mediates stress in plants, the data presented here suggest that exposing plants to at least mild hypobaric conditions can also serve to mediate plant stress in low oxygen environments. Interestingly, although the importance of oxygen in human hypobaric stress, such as the high-altitude pulmonary edema common to extreme mountain climbers, has long been appreciated (Hackett and Roach, 1987), it is becoming increasingly clear that the combination of stresses associated with hypobaric environments elicits complex physiological responses, and requires complex mediation (Bhagi et al., 2014). The establishment of protected agriculture in extreme environments—whether on the surface of Mars, in exploration vehicles, or for terrestrial applications—will require careful management of *in situ* resources and engineering considerations to optimize the internal environment for plant health and productivity. For instance, higher gas pressure differentials between inside and outside of greenhouse structures significantly increases the leak rate and therefore any reduction of internal pressure will bring engineering benefits. Further, the ability to lower specific components of the gas mixture to grow plants would bring a benefit of reduced transportation of gases and increased payload. However, regardless of the engineering benefits, it is imperative that the biological consequences of hypobaric environments be well understood before adopting this strategy for exploration life support habitats.

### Corroboration of array data using qRT-PCR

The FDR adjustment of *p*-value in our data would hide all differentially expressed genes in some comparisons such as 50 kPa responses. The *p*-value without correction helped us to detect differentially expressed genes. Two genes with *p* < 0.01 but with FDR > 0.05 in the arrays were used to evaluate the differential expression with qRT-PCR. AHB1 (AT2G16060) and PDC1 (AT4G33070) were induced by 50 kPa in roots. The values of Log2 fold-change of these two genes based on 97 kPa control were shown in Figure S2. Quantification with qRT-PCR showed that AHB1 and PDC1 were both significantly up-regulated in roots in response to 50 kPa, 25 kPa, and 97 kPa/HypOx, which was consistent with array results (Figure S2). These results support the conclusion that FDR adjustment limited the identification of genes significantly responding to hypobaric conditions, and that using *p* < 0.01 without correction can help reveal these responsive genes.

## Conclusions

The data presented here show that plant physiological adaptation to hypobaria is certainly more involved than a simple response to hypoxia. Based on the patterns of gene expression in hypobaria with supplemental oxygen, the most influential feature of hypobaria beyond hypoxia is a water stress apparently associated with the overall reduction of gas pressure, which manifests as mimicked aspects of drought stress. However, gene expression patterns also suggest that a plant's response to hypobaria is more complex than simply a combination of hypoxia and drought stress. The gene expression patterns of plants in a normoxic low pressure environment suggest that plants are engaging genes associated with a complex stimulus that induces a novel metabolic pattern involving multiple unknown signaling components that is distinct from effects derived from exposure to either hypoxia or drought. Further, these data revealed that the molecular responses are organ specific in that roots and shoots each possess distinct metabolic strategies for adjusting to hypobaria. Taken together these data suggest that while the stress response to the novel environment of hypobaria can be largely understood as an appropriate response to the component stresses of hypoxia and water loss, those two components do not fully explain responses to hypobaria. It is likely that in a long term scenario, such as a hypobaric greenhouse, much of the metabolic stress could be ameliorated simply by supplying sufficient water to offset an increase in evapotranspiration. In addition, plant line selection and genetic engineering could also contribute to developing plants better able to thrive in extended hypobaric environments.

## Author contributions

AP and RF contributed equally, and were responsible for the overall experimental design and conduct of the experiments. MZ performed the data analysis and took the lead on manuscript development. JC, MR, and MS prepared plant materials and conducted the low atmospheric pressure treatments. AR carried out statistical analysis of array data. AZ contributed to data analyses and was responsible data archiving in GEO. MD is the director of the University of Guelph Controlled Environment Systems Research Facility, and contributed to the experimental design. All authors read and approved the final manuscript.

### Conflict of interest statement

The authors declare that the research was conducted in the absence of any commercial or financial relationships that could be construed as a potential conflict of interest.

## Supplementary material

Figure S1 **Differentially expressed genes in response to 50 kPa/NormOx (50 kPa/pO_**2**_ = 21 kPa) and 25 kPa/NormOx (25 kPa/pO_**2**_ = 21 kPa) in roots and shoots of 10 d plants**. There are 40 genes showing statistically significant (*p* < 0.01) differential expression by at least 2-fold in at least in one of 50 kPa/NormOx and 25 kPa/NormOx in roots, and 3 genes in shoots. Heat map was graphed according to log value of fold change. Fill colors correspond to Figure 5A.

Figure S2 **Confirmation of gene expression profiles using qRT-PCR**. The transcript levels of AHB1 (AT2G16060) and PDC1 (AT4G33070) were determined by Taqman quantitative RT-PCR for RNA samples from the same 10 d roots tissues used for microarray analysis and additional experimental replications. The UBQ11 (AT4G05050) was used as the internal control. The Log2 fold-change of expression level relative to 97 kPa control for each sample was shown. Data are means ± SE (*n* = 3). Color bars represent Log2 fold-change in microarray data. Filled colors correspond to Figure 5A.

Table S1 **Full list of differentially expressed genes in response to 50 kPa and 50 kPa/NormOx (50 kPa/pO_**2**_ = 21 kPa) in roots and shoots of 10 d plants**. There are 151 genes that present significant (*p* < 0.01) differential expression by at least 2-fold in at least one condition (including responses to 50 kPa and 50 kPa/NormOx in roots or shoots) in 10 d plants. The differentially expressed genes are categorized according to log value of fold change and GO terms of biological process are listed for each gene clade.

Table S2 **Full list of differentially expressed genes in response to 50 kPa and 50 kPa/NormOx (50 kPa/pO_**2**_ = 21 kPa) in roots of 10 d and 5 d plants**. In a comparison between 10 and 5 d plants, 221 genes show statistically significant (*p* < 0.01) differential expression by at least 2-fold in at least one condition (in response to 50 kPa and 50 kPa/NormOx in roots of 10 d or 5 d plants). The differentially expressed genes are categorized according to log value of fold change and GO terms of biological process are listed for each gene clade.

Table S3 **Full list of differentially expressed genes in response to 25 kPa, 25 kPa/NormOx (25 kPa/pO_**2**_ = 21 kPa), and 97 kPa/HypOx (97 kPa/pO_**2**_ = 5 kPa) in roots and shoots of 10 d plants**. There are 372 genes showing statistically significant (*p* < 0.01) differential expression by at least 2-fold in at least one condition—including responses to 25 kPa, 25 kPa/NormOx, and 97 kPa/HypOx in roots and shoots. The differentially expressed genes are categorized according to cluster through log value of fold change and GO terms of biological process are listed for each gene clade. Among these, there are 93 genes showing differential expression by at least 1.8-fold between responses to 25 kPa and 25 kPa/NormOx, and 5 genes in comparison between 25 kPa and 97 kPa/HypOx in roots. For shoots, there are 44 and 11 genes in comparison between 25 kPa and 25 kPa/NormOx and between 25 kPa and 97 kPa/HypOx, respectively. For core hypoxia genes, 34 of them are included in 372 differentially expressed genes associated with 25 kPa atmosphere.

Table S4 **List of genes in pathway enrichment**. The differentially expressed genes mapped to KEGG pathways in the enrichment analysis (Benjamini corrected *p* < 0.05) were listed. Responses include 50 kPa, 50 kPa/NormOx (50 kPa/pO_2_ = 21 kPa), 25 kPa, 25 kPa/NormOx (25 kPa/pO_2_ = 21 kPa), and 97 kPa/HypOx (97 kPa/pO_2_ = 5 kPa) in roots and shoots of 10 d plants.
