# Supplementary figures and images for: Dissecting Low Atmospheric Pressure Stress: Transcriptome Responses to the Components of Hypobaria in Arabidopsis
Source: Front Plant Sci. 2017 Apr 10;8:528. doi: 10.3389/fpls.2017.00528 (PMC5385376; doi:10.3389/fpls.2017.00528)

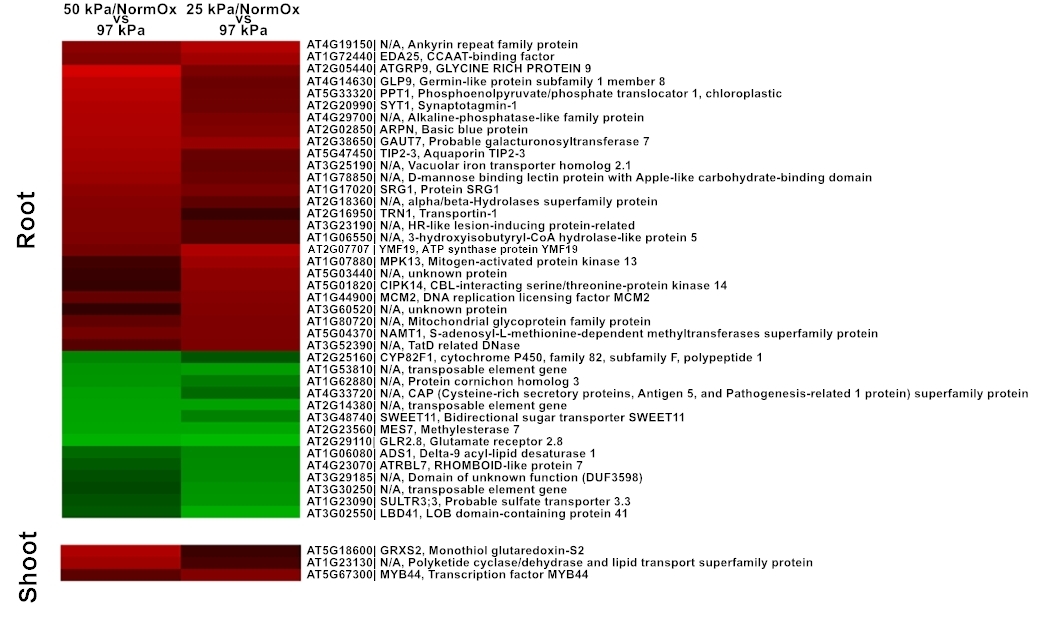

Supplement: Figure S1 — Differentially expressed genes in response to 50 kPa/NormOx (50 kPa/pO2 = 21 kPa) and 25 kPa/NormOx (25 kPa/pO2 = 21 kPa) in roots and shoots of 10 d plants. There are 40 genes showing statistically significant (p < 0.01) differential expression by at least 2-fold in at least in one of 50 kPa/NormOx and 25 kPa/NormOx in roots, and 3 genes in shoots. Heat map was graphed according to log value of fold change. Fill colors correspond to Figure 5A. [file Image1.JPEG]

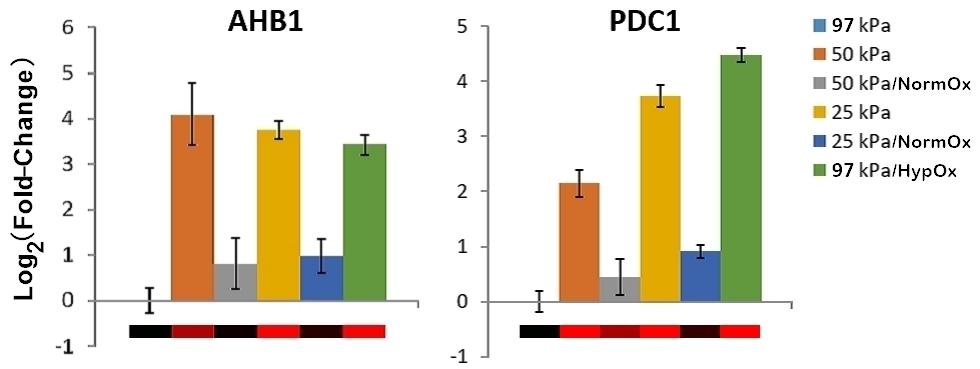

Supplement: Figure S2 — Confirmation of gene expression profiles using qRT-PCR. The transcript levels of AHB1 (AT2G16060) and PDC1 (AT4G33070) were determined by Taqman quantitative RT-PCR for RNA samples from the same 10 d roots tissues used for microarray analysis and additional experimental replications. The UBQ11 (AT4G05050) was used as the internal control. The Log2 fold-change of expression level relative to 97 kPa control for each sample was shown. Data are means ± SE (n = 3). Color bars represent Log2 fold-change in microarray data. Filled colors correspond to Figure 5A. [file Image2.JPEG]
